# Multi-Drug-Resistant Tuberculosis Peritonitis: A Case Report

**DOI:** 10.7759/cureus.53975

**Published:** 2024-02-10

**Authors:** Rhea Verma, Clinton Sonier, Nida Rizvi, Rahul Kashyap

**Affiliations:** 1 Medicine, Drexel University College of Medicine, Philadelphia, USA; 2 Internal Medicine, WellSpan Health, York, USA; 3 Research, Global Remote Research Program, St Paul, USA; 4 Critical Care Medicine, Mayo Clinic, Rochester, USA; 5 Research, WellSpan Health, York, USA

**Keywords:** iris, hiv, multi-drug resistant, ascites, peritonitis, tuberculosis

## Abstract

The increasing incidence of tuberculosis raises concerns globally, impacting both developing and developed nations. Abdominal tuberculosis stands out as the most prevalent form of extrapulmonary tuberculosis. This case report details the diagnostic journey of a young male with abdominal TB complicated by concurrent HIV infection. The patient presented with night sweats and substantial weight loss, concurrently receiving a naive human immunodeficiency virus (HIV) diagnosis with an undetectable CD4 count. Imaging revealed abdominal lymphadenopathy concealing the pancreatic head while bronchoscopy unveiled TB in the lung. The patient faced septic shock and bilateral pulmonary embolism, possibly due to immune reconstitution inflammatory syndrome (IRIS). The patient then developed ascites, and a diagnosis of TB peritonitis was made based on low serum ascites albumin gradient (SAAG) and a positive acid-fast bacillus (AFB) result in the para-aortic lymph node. Treatment complexity arose from drug resistance to isoniazid and ethambutol.

## Introduction

Abdominal tuberculosis (TB) is one of the most common forms of extrapulmonary TB; however, diagnosing patients with this condition is often challenging [1]. The absence of specific clinical features and the limited sensitivity of diagnostic tests frequently contribute to difficulties in timely identification. Symptoms of abdominal TB, such as abdominal pain and distention, can mimic conditions such as Crohn's disease, postoperative granulomatous peritonitis, and other malignancies of the abdominal organs, complicating the diagnostic process [2,3]. Without a high degree of suspicion, a diagnosis can easily be missed or delayed. Several risk factors can render individuals susceptible to TB infection, including being human immunodeficiency virus (HIV)-positive, which increases vulnerability to opportunistic infections. Co-infected individuals with both HIV and TB face increased risks of both diseases progressing rapidly, leading to higher mortality rates if not promptly diagnosed and effectively treated [4]. We aim to demonstrate how tuberculosis peritonitis with HIV co-infection can present diagnostic, therapeutic, and prognostic challenges not commonly encountered in clinical practice, highlighting the need for a high degree of suspicion for timely recognition and intervention.

## Case presentation

**Figure 1:**
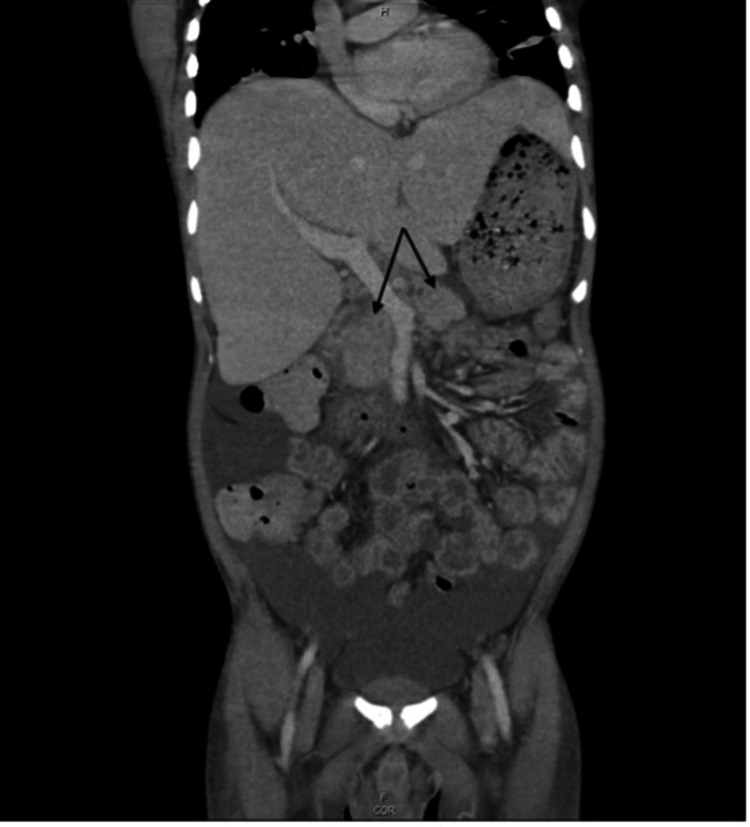
CT of the abdomen and pelvis with contrast, coronal view There is diffuse ascites throughout the abdomen. Arrows from left to right indicate necrotic para-aortic and celiac lymph nodes.

A male in his 30s recently relocated from Haiti to the United States with no known medical history. On January 29, 2023 (Day 0), he presented to the emergency department with abdominal pain, fever, a significant 100 lb weight loss, and night sweats. Notably, he had no cough, shortness of breath, or known tuberculosis exposures. CT imaging revealed features of acute pancreatitis, including possible pancreatic head necrosis and mesentery inflammation (Figure 1). MRI showed extensive abdominal lymphadenopathy, complicating visualization of the pancreatic head. Thrush was observed during the physical examination, leading to a new diagnosis of HIV with an undetectable CD4 count. To further investigate the cause of his symptoms, a bronchoscopy was indicated, which confirmed TB in the right lower lung. His left cervical lymph node was also found to be positive for acid-fast bacilli (AFB), indicating disseminated tuberculosis (TB). This finding prompted the initiation of TB treatment including IV Amikacin, Azithromycin, Rifampin, Ethambutol, Isoniazid, and Pyrazinamide. Shortly after the initiation of TB treatment (Day 10), antiretroviral therapy (Truvada and Tivicay) was also started. The CD4 count improved to over 250 with treatment.

**Figure 2:**
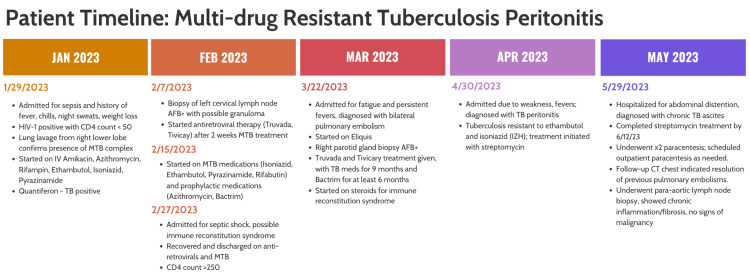
Graphical representation illustrating the timeline of important events over multiple hospitalizations

The patient experienced septic shock on February 27, 2023 (Day 29), possibly due to immune reconstitution inflammatory syndrome (IRIS), which was managed successfully with antibiotics, steroids, and midodrine. Three weeks after that (Day 52), he was admitted for bilateral pulmonary embolism (PE) with unknown etiology but likely multifactorial due to AIDS, disseminated TB, and peripancreatic lymphadenopathy. Finally, on April 30, 2023 (Day 91), the patient developed ascites (Figure 2). The patient underwent a paracentesis, and despite the AFB culture being negative, the diagnosis of TB was supported by a positive AFB result in the para-aortic lymph node. A low serum ascites albumin gradient (SAAG) value also suggested that liver-related causes were less likely. Drug sensitivity testing revealed resistance to isoniazid and ethambutol but sensitivity to streptomycin, pyrazinamide, and rifabutin. Therapeutic paracentesis was performed as needed, and the patient improved with alternative TB treatment (Table 1).

**Table 1:** Summary of biological findings: blood tests, imaging studies, microbiology, and other clinical observations

Category	Date	Test/Parameter	Result
Blood Tests	Day 0	HIV Antibody Test	Positive
	Day 0	CD4 Count	Undetectable
	Day 91	Drug Sensitivity Testing	Resistant to isoniazid and ethambutol, sensitive to streptomycin, pyrazinamide, and rifabutin
Imaging Studies	Day 0	CT Imaging	Features of acute pancreatitis, possible pancreatic head necrosis, and mesentery inflammation
	Day 0	MRI	Extensive abdominal lymphadenopathy, poor visualization of the pancreatic head
	Day 52	CT Pulmonary Angiography	Bilateral pulmonary embolism
Microbiology	Day 0	Bronchoscopy	TB in the right lower lung
	Day 0	Lymph Node Biopsy	Positive acid-fast bacilli in the left cervical lymph node
Other	Day 0	Clinical assessment	Abdominal pain, fever, significant weight loss, night sweats, and thrush
	Day 29	Clinical assessment	Septic shock managed with antibiotics, steroids, and midodrine
	Day 91	Clinical assessment	Development of ascites, therapeutic paracentesis performed as needed

By May 29, 2023, the patient was doing well, gained weight, and no longer required therapeutic paracentesis. As of January 2024, the patient continues to do well, with completed TB treatment and well-controlled HIV. No residual issues have arisen since.

## Discussion

Although rare, tuberculosis peritonitis cases continue to be reported in the United States and other developed nations. Among individuals born outside the United States, the case rate is 13 times higher compared to those born in the country, where the rate remains extremely low, at only 1.2 cases per 100,000 [5]. The risk factors for TB peritonitis include the occurrence of immunocompromised states, including human immunodeficiency virus (HIV) infection and alcoholic liver disease, along with an increase in migration from regions where tuberculosis is common [6]. Approximately 50% of individuals with HIV/AIDS experience extrapulmonary symptoms of tuberculosis while only 10-15% of TB patients without HIV develop extrapulmonary symptoms [6]. Accordingly, our patient's immigration history from Haiti was concerning for TB. The HIV diagnosis further heightened the risk, as individuals with HIV/AIDS are more prone to the dissemination of tuberculosis.

This patient's case exemplifies the challenges in diagnosing TB peritonitis with HIV co-infection, further complicated by drug resistance. In those with both HIV and TB, the symptoms of abdominal TB, such as vomiting, abdominal pain, ascites, weight loss, and fever, are nonspecific and may be easily mistaken for other gastrointestinal conditions. Previous case series on HIV and TB peritonitis reveal atypical characteristics in tuberculosis peritonitis, deviating significantly from classical presentations. These cases also demonstrate notably suboptimal responses to therapy, resulting in an unfavorable prognosis [7-9].

HIV patients with active tuberculosis not only experience a higher incidence of new AIDS-defining opportunistic infections but also face a shorter overall survival compared to HIV-infected patients without tuberculosis [10]. The co-infection appears to be linked to an increased risk of death, even after considering factors such as age, intravenous drug use, previous opportunistic infections, baseline CD4+ count, and antiretroviral therapy use.

Additionally, in people co-infected with HIV and TB, the risk of developing tuberculosis-immune reconstitution inflammatory syndrome (IRIS) is significant [11]. The pathogenesis of TB-IRIS remains incompletely understood, but certain clinical risk factors have been associated with its development. Patients with low CD4+ T cell counts at the start of antiretroviral therapy (ART), followed by a rapid increase in CD4 counts after ART, are more susceptible to TB-IRIS due to an exaggerated T cell response to Mycobacterium (M.) tuberculosis and the overproduction of inflammatory cytokines [12]. Other risk factors include a short interval between starting antitubercular therapy and ART, dissemination of TB infection to extrapulmonary organs, and high HIV-1 viral load [11]. Considering these factors can be crucial when deciding the optimal time to initiate ART in individuals co-infected with HIV and TB and may have played a role in the pathogenesis of TB-IRIS in our patient's case.

Another complicating factor in this patient’s journey was the drug-resistance properties of the TB strain. The strain was found to be resistant to isoniazid (INH) and ethambutol, which are commonly used first-line drugs for TB treatment [13]. Instead, it was sensitive to streptomycin, pyrazinamide, and rifabutin. Tuberculosis drug resistance, especially to drugs like isoniazid and ethambutol, can result from genetic mutations, drug interactions, improper antibiotic use, and patient non-adherence. Regularly monitoring how the treatment works and periodically reassessing drug susceptibility is crucial for adjusting the treatment plan as needed. This case highlights the variability in drug resistance patterns among individuals with TB, emphasizing the need for individualized and targeted therapeutic approaches, especially in the context of coexisting conditions, such as HIV, and complications like peritonitis.

## Conclusions

This case illustrates the diagnostic complexities associated with uncommon tuberculosis presentations, especially in individuals with co-infections like HIV. The added challenge of drug resistance emphasizes the need for personalized treatment approaches. This patient's journey highlights the critical role of comprehensive care, considering factors like immunological status, medication resistance, and potential immune-related reactions. Our case demonstrates the need for clinicians to maintain a high degree of suspicion for TB peritonitis, prompting further clinical investigation.
